# Untargeted Metabolomics of Xylem Sap Exudates in Two Common Bean Genotypes with Contrasting Growth Rates Under Water Deficit During Pod Filling

**DOI:** 10.3390/metabo16080564

**Published:** 2026-08-10

**Authors:** Norma Cecilia Morales-Elias, Lizandro Ramírez-Trejo, Carlos Alberto Cruz-Cruz, Juan Luis Monribot-Villanueva, José Antonio Guerrero-Analco, Monserrat Vázquez-Sánchez, José Rodolfo García-Nava, Antonio García-Esteva, María Teresa González-Arnao, José Cruz Jiménez Galindo, Daniel Padilla-Chacón

**Affiliations:** 1 Programa de Posgrado en Botánica, Colegio de Postgraduados, Campus Montecillo, Texcoco 56264, Mexico; morales.norma@colpos.mx (N.C.M.-E.); vazquez.monserrat@colpos.mx (M.V.-S.); garcianr@colpos.mx (J.R.G.-N.); esteva@colpos.mx (A.G.-E.); 2 Facultad de Ciencias Químicas, Universidad Veracruzana, Prolongación de Oriente 6, No. 1009, Orizaba 94340, Mexico; zs23000138@estudiantes.uv.mx (L.R.-T.); calcruz@uv.mx (C.A.C.-C.); teregonzalez@uv.mx (M.T.G.-A.); 3 Red de Estudios Moleculares Avanzados (REMAV), Instituto de Ecología A. C., Carretera Antigua a Coatepec 351, Xalapa 91070, Mexico; juan.monribot@inecol.mx (J.L.M.-V.); joseantonio.guerrero@inecol.mx (J.A.G.-A.); 4 Grupo de Genética y Mejora de Frijol, Instituto Nacional de Investigaciones Forestales, Agrícolas y Pecuarias, Campo Experimental Valle del Guadiana, Km 4.5, Carretera Durango-Mezquital, Durango 34170, Mexico; jimenez.cruz@inifap.gob.mx; 5 Secretaría de Ciencia, Humanidades, Tecnología e Innovación (SECIHTI), Facultad de Ciencias Químicas, Universidad Veracruzana, Orizaba 94340, Mexico

**Keywords:** carbon allocation, growth rate, *Phaseolus vulgaris*, xylem sap exudates, untargeted metabolomics, water deficit

## Abstract

**Highlights:**

**What are the main findings?**

- Water deficit during pod filling was associated with changes in the xylem sap metabolome, including flavonoids, isoflavonoids, glycosylated triterpenoid saponins, and other stress-related metabolites.
- The late-maturing genotype OTI showed a broader metabolic response than the early-maturing genotype RB under water deficit, with relatively higher levels of citrate, aromatic amino acids, tridecanedioic acid, and flavonoid-related metabolites.

**What are the implications of the main findings?**

- These genotype-specific metabolic responses may reflect different physiological strategies associated with water deficit during reproductive development.
- The metabolites and metabolic pathways identified in this study may provide a basis for future research on drought adaptation and the identification of candidate metabolic markers in common bean.

**Abstract:**

**Background/Objectives**: Water deficit during the pod filling stage severely limits the productivity of common beans (*Phaseolus vulgaris*). Although the effects of water scarcity have been extensively studied in leaves and roots, the contribution of xylem sap to systemic metabolic adaptation remains poorly understood. This study investigated genotype-specific metabolic changes in xylem sap exudates in response to water deficit in two common bean genotypes with contrasting growth rates. The OTI genotype has a growth cycle of 120 days, while Rosa La Bufa (RB) completes its cycle in 80 days under both well-watered and water deficit conditions. **Methods**: Physiological responses were evaluated, and xylem sap exudate metabolites were profiled using untargeted UPLC–ESI–QTOF–MS. Protein–metabolite interaction networks were reconstructed using STITCH v5 to identify genotype-specific metabolic organization under stress. **Results**: Water deficit reduced the abundance of multiple xylem metabolites, including flavonoids, isoflavones, phenolic acids, sugars, amino acids, and oxylipin-related compounds, indicating systemic metabolic contraction. OTI exhibited extensive metabolic changes characterized by enrichment of citrate, aromatic amino acids, glycolytic intermediates, flavonoid glycosides, and detoxification-associated metabolites, consistent with active carbon remobilization and oxidative stress responses. In contrast, RB accumulated isoflavones, including genistein, biochanin A, and baicalein, together with glycosylated triterpenoid saponins. Network analysis revealed that OTI developed a broad stress-responsive interactome that integrated phenylpropanoid metabolism, carbon remobilization, and detoxification pathways, whereas RB maintained a compact interactome reinforced by oxylipin-, jasmonate-, and isoflavone-associated modules. **Conclusions**: Xylem sap undergoes genotype-dependent metabolic adjustment under water deficit, suggesting contrasting drought adaptation strategies. Long growth cycle genotypes activate extensive metabolic plasticity, whereas reduced growth cycle genotypes rely on more specialized defense networks. These findings highlight xylem metabolomics as a valuable approach for understanding systemic drought adaptation and identifying putative metabolic biomarkers associated with drought resilience in common beans.

## 1. Introduction

Drought is the primary constraint affecting common beans (*Phaseolus vulgaris*) [1]. In Mexico, approximately 86–88% of common bean cultivation relies on rainfed agriculture [1,2]. The impact of water deficit depends on its duration, severity, developmental plant stage, and genotype [3]. Geneticists have developed breeding programs aimed at improving plant drought tolerance to mitigate the effects of climate change, particularly in regions with limited water availability [4]. Thus, genotypes have been developed through mechanisms of drought avoidance, escape, and tolerance, with trait expression varying according to the maturation stage [5]. For example, genotypes with a 120-day growth rate are generally categorized as tolerant to terminal drought stress, particularly during post-fertilization and flower set, supporting optimal pod maturation and improved yield stability. In contrast, earlier genotypes (70–80 days) tend to cope with drought through escape strategies, such as rapidly allocating water and nutrients to reproductive structures to avoid terminal stress [6].

Xylem is the tissue of vascular plants that transports water and nutrients from the soil to the stems and leaves and provides mechanical support and storage [7]. Techniques have been developed to extract xylem sap from stems or roots [8], which contain mostly inorganic compounds and minerals, as well as small amounts of amino acids, sugars, and organic acids [9,10], key for cellular turgor pressure, signaling, nutrient transport, and overall hydraulic functionality [11,12]. In particular, the xylem sap of field-grown legumes carries N-containing compounds originating from nodules as assimilation products of N_2_ fixation and soil mineral N taken up by the roots [13]. In many tropical and sub-tropical legumes, the main product of N_2_ fixation exported from the nodules is in the form of allantoin and allantoic acid (ureides) [14]. In these same species, solutes derived from soil mineral N are transported in the xylem as nitrate or as organic products of nitrate reduction (amino acids) [15].

In common beans, early studies demonstrated that xylem sap composition includes cytokinins as key regulators of plant growth and development [16]. Additionally, ref. [17] reported that cytokinin concentrations can increase by up to 400% within 24 h following decapitation and auxin application, highlighting the dynamic nature of xylem-mediated hormonal signaling. Recent studies have shown that xylem sap composition is closely associated with plant responses to drought stress [18,19,20,21,22]. Evidence indicates that water deficits alter sap flow dynamics and nutrient uptake efficiency, ultimately modifying xylem sap composition [20,23]. For instance, in maize, drought stress induces significant changes in phytohormones, such as abscisic acid (ABA) and cytokinins, as well as the accumulation of metabolites, including the aromatic cytokinin 6-benzylaminopurine (BAP) and other compounds associated with increasing drought severity [24]. These findings highlight the role of xylem sap as a dynamic signaling medium that integrates hormonal and metabolic responses under water deficit conditions.

Recently, gas chromatography–mass spectrometry (GC-MS), liquid chromatography–mass spectrometry (LC-MS), and nuclear magnetic resonance spectroscopy (NMR) have become important tools for investigating complex traits by linking metabolic changes to specific physiological responses [25,26]. These techniques have been used to characterize changes in sap composition and signals in root exudates of cotton [25,27], polyphenols in leaves of coffee plants [28], and to explore resistance to abiotic stress [29]. In poplar plants, drought enhances xylem sap sulfate, causing stomatal closure [30]. In addition, metabolomics of *Brassica juncea* xylem sap treated with cadmium (Cd) revealed significant differences in metabolic profiles. Differential metabolites are primarily involved in amino acids, organic acids, lipids, and carbohydrates, and most were down-accumulated, playing essential roles in response to Cd stress [31].

However, very limited information is available regarding the metabolic composition of xylem sap in common beans genotypes and their physiological relationship with plant adaptation to future water stress scenarios [32,33]. We hypothesized that the xylem metabolome is primarily structured by genotype under well-watered conditions, or that drought acts as the main driver of metabolic reconfiguration, remodeling specific pathways depending on the genetic background. Together, these findings suggest that drought-induced metabolic reprogramming in xylem sap may play a central role in coordinating genotype-dependent physiological responses under water deficit conditions. This work aims to understand how vascular metabolic plasticity contributes to contrasting water deficit response strategies in common bean.

## 2. Materials and Methods

### 2.1. Plant Material and Growth Conditions

Two common bean (*P. vulgaris*) genotypes, OTI and RB, were used in this study. OTI is a cultivar released for the Valley of Mexico by ref. [34], whereas Rosa La Bufa (RB), also referred to as Rosa Bufa in some of the literature, is an experimental genotype provided by the Instituto Nacional de Investigaciones Forestales, Agrícolas y Pecuarias (INIFAP) and previously evaluated under contrasting moisture conditions in Northern Mexico [35].

Both genotypes have previously been identified as drought tolerant; however, they differ in growth rate, growth habit, crop cycle, and physiological performance under water deficit [35,36]. OTI exhibits a determinate growth habit (Type I) with a crop cycle of approximately 110–130 days, while RB exhibits an indeterminate growth habit (Type II) with a shorter crop cycle of 60–80 days.

The experiment was conducted in a tunnel-type greenhouse during the summer growing season of 2023. The greenhouse was located at the experimental fields of Colegio de Postgraduados, Campus Montecillo, Texcoco, Estado de México, Mexico (19°27′40″ N, 98°54′19″ W; 2353 m a.s.l.).

### 2.2. Experimental Design and Crop Management

Fifty seeds of each genotype (OTI and RB) were sown individually into 5 L pots filled with 4 kg of agricultural soil, with one plant per pot constituting a single experimental unit in a completely randomized design. All plants were irrigated daily at 100% field capacity (FC) until the R8 (pod-filling) stage (Figure S1). This phenological classification was assigned according to the phenological scale for common beans proposed by the International Center for Tropical Agriculture (CIAT).

Thereafter, plants were randomly assigned to one of two soil moisture regimes, either as 100% FC (Control) or 50% FC assigned as water deficit (WD), and were maintained using a gravimetric method. This resulted in four treatment combinations: OTI-Control, OTI-WD, RB-Control, and RB-WD. The nutrient supply was standardized across treatments through the application of a granular NPK fertilizer (YaraMila^®^ 12–11–18 with micronutrients; Yara International ASA, Oslo, Norway).

### 2.3. Physiological Measurements

To evaluate the effects of water deficit, leaf water potential (Ψ) was measured daily using the pressure chamber method previously described by Scholander et al. (1965) [37]. Briefly, three fully expanded leaves were collected from the same positions (middle canopy) of plants from the OTI-Control, OTI-WD, RB-Control and RB-WD treatments, and the Ψ was recorded using a Scholander-type pressure chamber (PMS Instrument Company, Model 15D15T, Albany, OR, USA). Compressed nitrogen gas was used to gradually increase the pressure inside the chamber until xylem sap became visible at the cut surface of the petiole, and the balancing pressure (MPa) was recorded as the leaf water potential. Measurements were conducted at 10:00 h (mid-morning) to minimize diurnal variation in leaf water status, using three independent biological replicates per treatment (*n* = 3).

### 2.4. Agronomic and Yield Traits

Based on evidence suggesting that xylem sap may contain growth-related signals, the effects of water deficit were evaluated on yield-related traits at the R9 or full maturity stage. Three individual plants per treatment (OTI-Control, OTI-WD, RB-Control, and RB-WD) were used as biological replicates (*n* = 3). The evaluated traits included seed mass (g plant^−1^), number of pods per plant, number of seeds per pod, and 100-seed weight per treatment.

### 2.5. Xylem Sap Collection

Xylem sap was collected from the basal stem of common bean plants after 10 days of water deficit treatment (50% field capacity [FC]) or under well-watered control conditions (100% FC) at the R8 stage (pod filling). Xylem sap was sampled between 10:00 and 11:00 h to minimize diurnal variation in its composition excising the entire plant and immediately placing the cut basal stem into a Scholander-type pressure chamber (PMS Instrument Company, Model 15D15T, Albany, OR, USA) connected to a nitrogen gas cylinder, following the protocol described by [8] with minor modifications.

Before sap extraction, the cut surface of the basal stem was gently rinsed with sterile distilled water to remove potential surface contaminants. Chamber pressure was gradually increased to a maximum of approximately 0.5 bar min^−1^ gradually increased to a maximum of 35 bar until xylem sap droplets appeared at the cut surface. To minimize contamination from damaged phloem tissues, the initial droplets were discarded using sterile absorbent paper. Subsequent xylem sap exudates were collected using micropipettes and transferred into sterile, pre-chilled 1.5 mL microcentrifuge tubes over approximately 3 min per plant, which continued until sap flow ceased or a sufficient volume was obtained.

For each genotype and irrigation treatment, xylem sap was collected from five independent plants and pooled to generate a single biological composite sample. Pooling ensured sufficient sap volume for untargeted metabolomic analysis while reducing the influence of transient plant-to-plant physiological variation on the metabolite profile. Four pooled samples were obtained: (1) OTI-Control, (2) OTI-WD, (3) RB-Control, and (4) RB-WD. Approximately 4 mL of xylem sap was obtained for each pooled sample. Samples were immediately frozen in liquid nitrogen after collection and stored at −80 °C until lyophilization. Samples were stored at −80 °C until lyophilization. The lyophilized samples were subsequently used for metabolite extraction and UPLC-MS analysis.

### 2.6. Untargeted Metabolomic Analysis

For the identification of differential metabolites associated with water stress, untargeted metabolomic analyses were carried out as previously reported by ref. [38,39]. Crude extracts from xylem sap were obtained using an accelerated solvent extraction system (ASE350, Dionex, Thermo Scientific, Waltham, MA, USA) with 80% methanol (HPLC grade in MilliQ H_2_O) as extraction solvent, from which excess was further eliminated by rotary evaporation (Rotovap^®^ RII, Büchi, Newmarket, UK) until dryness. Extracts from all samples were dissolved at 20 mg mL^−1^ in 1 mL of 80% methanol with 0.1% of formic acid (all MS grade, Sigma-Aldrich, St. Louis, MO, USA), filtered using a 0.2 µm PTFE filters, and then placed in 1.5 mL UPLC. The samples were analyzed by technical triplicates in a randomized order. In addition, blank and pooled samples were injected as quality-control samples. Pooled samples were used to for peak detection and alignment, as well as in the deconvolution process. All signals that exhibited the highest intensity in the blank samples were excluded from the analysis. Analyses were performed on an ultra-high-performance liquid chromatography system (UPLC, Acquity class I, Waters™, Milford, MA, USA) coupled to a high-resolution quadrupole time-of-flight mass spectrometer (QTOF, Synapt G2-Si model, Waters™). Chromatographic separation was carried out on an Acquity BEH column (1.7 µm, 2.1 × 50 mm) at a temperature of 40 °C and 15 °C for each sample. The mobile phase consisted of (A) water and (B) acetonitrile, both with 0.1% formic acid. The conditions of the mobile phase gradient were 0–13 min linear gradient 1–80% B, 13–14 min 80% B isocratic, and 14–15 min linear gradient 80–1% B (total analysis time was 20 min). The flow rate was 0.3 mL min^−1^, and 5 µL of each extract was injected. Mass spectrometry analysis was performed with an electrospray ionization source in positive and negative modes. The capillary, sampling cone, and source offset voltages were 3000, 40, and 80 V, respectively. The source temperature was 120 °C, and the desolvation temperature was 300 °C. The desolvation gas flow was 650 L h^−1^, and the nebulizer pressure was 6.0 bar. The mass range was from 50 to 1200 Da, with function 1 CE of 6 V, function 2 CER of 10–30 V, and an exploration time of 0.5 s. Leucine–enkephalin (556.2771 [M + H]^+^; 554.2615 [M − H]^−^) was used as a reference solution. Mass/charge ratios (*m*/*z*), retention time (RT), and peak intensity (total counts) were obtained and analyzed using Masslynx (version 4.1, Waters™) and Progenesis QI (version 3.0, Nonlinear Dynamics) softwares.

### 2.7. Data Imputation, Transformation and Normalization

Metabolomic data acquired in positive and negative ionization modes were processed and analyzed separately using R v4.6 (R Core Team, 2026). For each ionization mode, missing and zero values were independently replaced with random values drawn from a uniform distribution bounded by the 1st and 5th percentiles of the non-zero intensities detected in each respective sample. Intensity values were then log_2_-transformed and subjected to quantile normalization, using the normalize between arrays function from the limma package v3.68.4 [40] to reduce technical variation and improve comparability across samples.

### 2.8. Metabolite Annotations and Differential Analysis

Each retained feature was hierarchically annotated using three reference metabolomic databases, prioritized in the following order: *Phaseolus vulgaris*, Fabaceae, and a curated list of nine biologically relevant metabolites. Only features that matched at least one annotation level were retained for downstream analyses.

Candidate metabolites were accepted when the mass error was within ±10 ppm and supported by isotope distribution matching and MS/MS fragmentation data. Annotation confidence was evaluated using the integrated score generated by Progenesis QI v3.0 (Nonlinear Dynamics, Newcastle, UK) software. Candidate annotations were ranked according to their annotation score. When two or more candidate metabolites presented similar scores (difference ≤ 2 units), feature abundance was used as a secondary selection criterion, and the candidate associated with the highest signal intensity was retained as the most probable annotation. No authentic reference standards were analyzed; therefore, all metabolite assignments should be considered as putative annotations (MSI Level 2). Complete annotation information, including retention time, *m*/*z* values, isotopic similarity, fragmentation scores, annotation scores, and adduct information, is provided in Table S1.

Differential abundance analysis was performed independently for each ionization mode using the limma package [40]. For each feature, a linear model was fitted using lmFit to evaluate three comparisons: OTI water deficit vs. control, RB water deficit vs. control, and an overall genotype comparison integrating both water treatments (OTI vs. RB). Statistical significance was assessed using empirical Bayes moderated t-statistics, incorporating intensity-dependent variance modeling and robust variance estimation to account for the mean–variance relationship and reduce the influence of outlier features. Raw *p*-values were adjusted using the Benjamini–Hochberg procedure to control the false discovery rate (FDR). Features were classified as differentially abundant when the FDR-adjusted *p*-value was <0.05 and the fold change was >1.5. Finally, results from both ionization modes were merged for downstream interpretation. Considering the pooled biological sampling strategy, these differentially abundant metabolites should be regarded as putative candidate biomarkers and interpreted as exploratory findings.

Metabolites with available compound names are shown by their common name, whereas metabolites with names exceeding 30 characters are displayed using their PubChem Compound Identifier (CID). Only the most statistically significant metabolites are labeled in the volcano plots (Table S2).

### 2.9. Multivariate and Clustering Analysis

To explore natural sample groupings and metabolic variability, an unsupervised principal component analysis (PCA) was performed using the FactoMineR v2.14 [41] and factoextra v2.0.0 R packages. A supervised Partial Least Squares Discriminant Analysis (PLS-DA) was conducted using the ropls package (v1.44.0) [42]. Metabolites with Variable Importance in Projection (VIP) scores > 1 were retained as candidate biomarkers. Model validity was evaluated by permutation testing (1000 permutations) based on the predictive performance (*Q*^2^) and goodness-of-fit (*R*^2^*Y*) statistics.

Relative abundance patterns were visualized using heatmaps, followed by hierarchical clustering analysis (HCA) of samples and metabolites using Euclidean distance and Ward.D2 linkage. The optimal number of clusters was determined using the elbow (WSS), silhouette, and gap statistic methods. Prior to all multivariate analyses, data were mean-centered and scaled.

### 2.10. Enrichment Analysis

Metabolic pathway enrichment analysis was performed using the Pathway Analysis module in MetaboAnalyst v6.0 [43], with the *Glycine max* metabolic pathway library used as the reference. Analyses were conducted separately for each comparison (OTI-WD vs. OTI-Control, RB-WD vs. RB-Control, and OTI vs. RB). Only metabolites identified with MSI Level 2 confidence and classified as differentially accumulated (FDR-adjusted *p* < 0.05 and fold change > 1.5) were included in the enrichment analyses. For drought-response comparisons, both significantly up- and down-accumulated metabolites were considered. For the genotype comparison, enrichment analysis was performed using metabolites that differed significantly between genotypes regardless of water treatment. Chemical similarity enrichment analysis was performed using ChemRICH [44]. KEGG pathway enrichment groups analysis in metabolic pathways was performed independently for each comparison using metabolites classified as differentially accumulated (FDR-adjusted *p* < 0.05 and fold change > 1.5). The horizontal axis represents the pathway significance as −log10 (*p*-value), the bubble size reflects the pathway impact, and the bubble color indicates the strength of enrichment (darker colors correspond to higher −log10 (*p*-value) values. Squares denote down-regulated pathways and triangles denote up-regulated pathways.

### 2.11. Protein–Metabolite Interaction Analysis

Functional association networks were constructed to explore the interactions between differentially accumulated metabolites and predicted protein targets. The STITCH database v.5.0 [45] was utilized, using *Arabidopsis thaliana* as the reference organism for protein mapping. To maintain focus on induced resilience mechanisms, the analysis was restricted exclusively to metabolites found to be significantly accumulated (fold change > 1.5 and FDR-adjusted *p* < 0.05) in four specific comparisons: OTI genotype putative candidate markers (OTI-Up), RB genotype putative candidate markers (RB-Up), and the drought-induced responses (OTI-WD-Up and RB-WD-Up).

## 3. Results

### 3.1. Soil Moisture Dynamics Under Water Deficit

**Figure 1:**
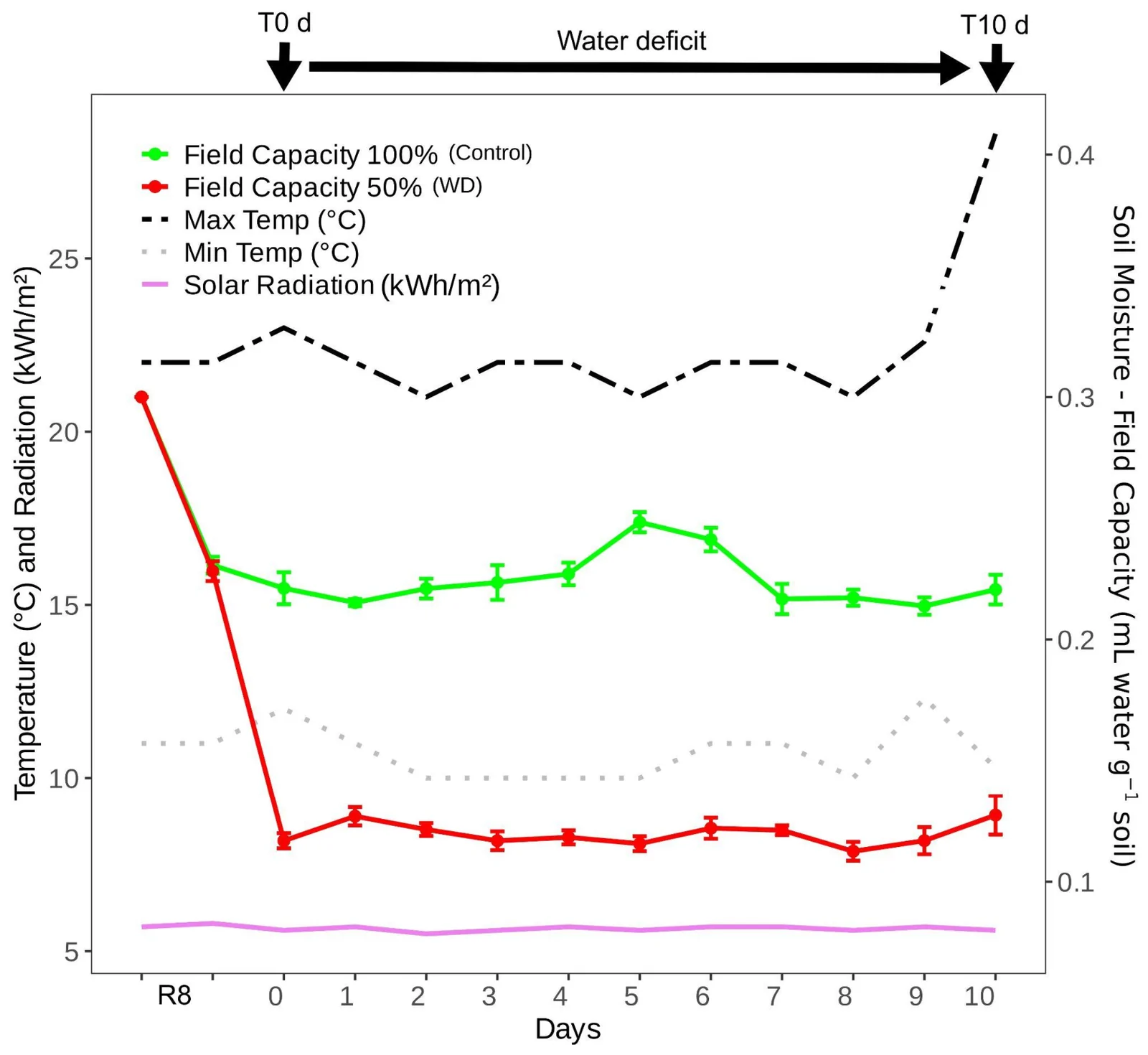
Soil moisture dynamics and environmental conditions during the 10-day water deficit period. Control = 100% FC; WD = 50% FC. Soil moisture (right axis) was monitored in plants maintained at 100 and 50% field capacity (FC) from the R8 reproductive stage (pod filling) to 10 days after the onset of water deficit. Maximum and minimum air temperatures and daily solar radiation (left axis) are also shown. Soil moisture values represent SE (*n* = 3). Water deficit was initiated at day 0 and maintained for 10 days.

The loss of soil water decreased rapidly as a result of withholding irrigation at 50% FC (Figure 1) during the first 3 days of stress imposition, with values ranking between 0.30 mL g^−1^ at 24 h, and further declined to 0.12–0.14 mL g^−1^ at 48 h, marking the onset (T0) of water deficit.

**Figure 2:**
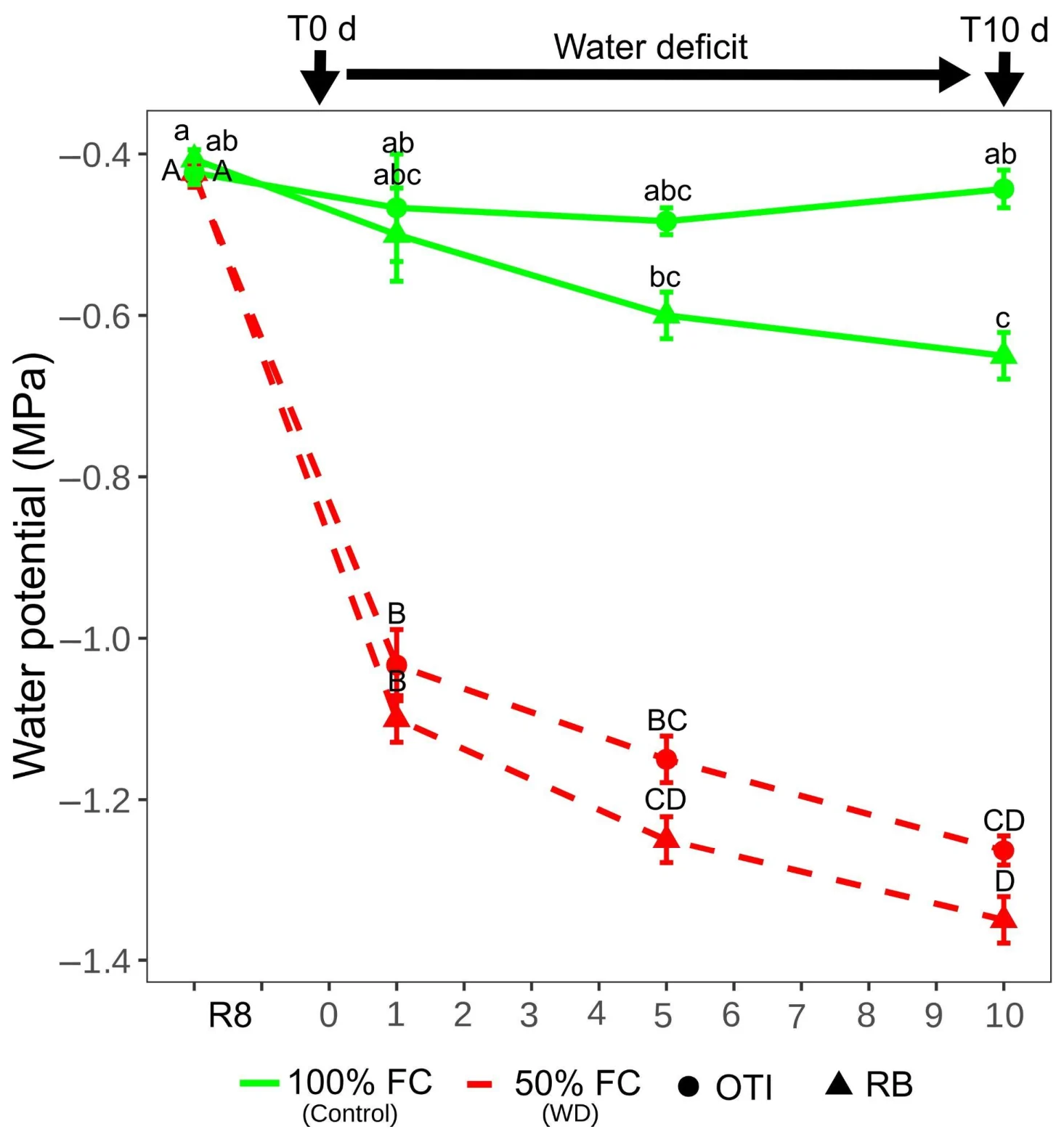
Leaf water potential (Ψ_leaf_) of OTI and RB plants subjected to 100 and 50% field capacity (FC) during a 10-day water deficit period. Water deficit began at day 0 (T0). Measurements were performed at R8 stage (pod filling) and at 0, 2, 4, 6, 8, and 10 days after treatment onset. Data represent means ± SE (*n* = 3). Different letters indicate significant differences among treatments according to ANOVA followed by Tukey’s test (*p* < 0.05). Solid lines indicate 100% FC while dashed lines indicate 50% FC. Circles correspond to OTI and triangles to RB.

This effect was consistent with the changes in leaf water potential (Ψ_leaf_) (Figure 2). At R8, Ψ_leaf_ values were similar across treatments and genotypes (−0.40 MPa). Plants maintained at 100% FC exhibited relatively stable Ψ_leaf_ values throughout the experimental period from −0.45 to −0.55 MPa. In contrast, under 50% FC, Ψ_leaf_ declined sharply to approximately −1.0 MPa at day 0 (T0) and continued to decrease to between −1.25 and −1.35 MPa by day 10 (*p* < 0.05).

**Figure 3:**
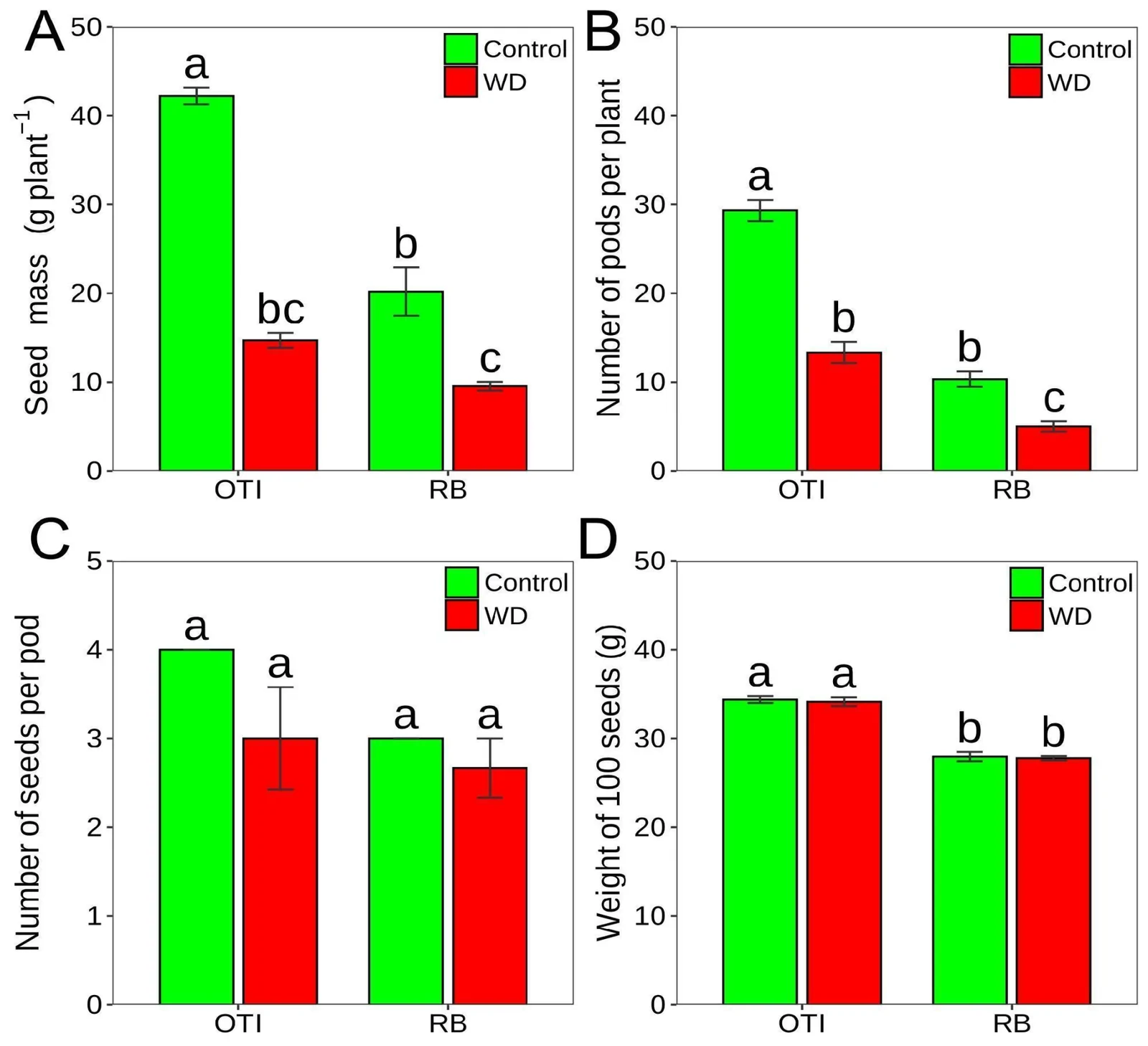
Yield components of OTI and RB plants grown under 100 and 50% field capacity (FC). (**A**) Grain yield per plant (g plant^−1^), (**B**) number of pods per plant, (**C**) number of seeds per pod, and (**D**) 100-seed weight (g). Values represent means ± SE (*n* = 3). Different letters indicate significant differences among treatments according to ANOVA followed by Tukey’s test (*p* < 0.05).

This reduction in plant water status was accompanied by changes in yield components (Figure 3). Under water deficit, both genotypes showed a significant increase in pod abortion (64–66%; Figure 3B). In contrast, the number of seeds per pod was not significantly affected by irrigation treatment (Figure 3C), and the 100-seed weight remained stable across treatments, with differences observed only between genotypes (Figure 3D).

### 3.2. Xylem Metabolome Response to Water Deficit

A total of 209 metabolites were putatively annotated (MSI Level 2) from the xylem sap metabolome (Table S1). The annotated compounds comprise amino acids, organic acids, carbohydrates, nucleosides, fatty acids, flavonoids, isoflavones, phenolic acids, glycosylated triterpenoid saponins, and other secondary metabolites. Complete annotation information is provided in Table S1.

**Figure 4:**
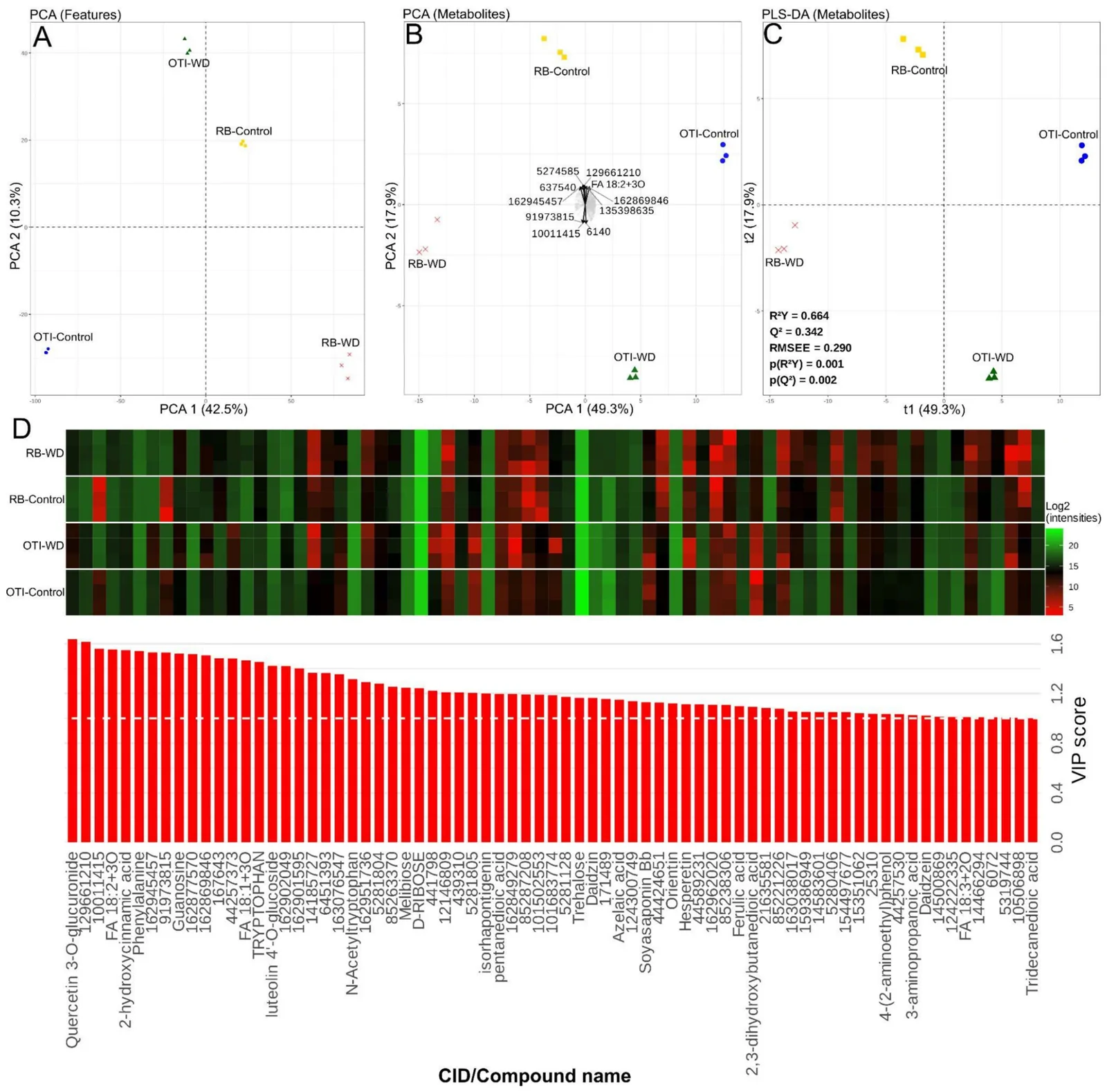
Multivariate analysis of xylem metabolome. (**A**,**B**) PCA of spectrometric features and annotated metabolites. (**C**) PLS-DA showing genotype and treatment separation. (**D**) Heatmap of relative abundance (Log_2_) and VIP scores of the top discriminant metabolites. In panels (**B**,**D**), labels correspond to PubChem Compound Identifiers (CIDs) of metabolites contributing most strongly to sample separation; metabolite identities are listed in Table S1. In panel (**B**), arrows indicate the metabolites with the highest loadings and greatest contribution to sample separation. In panel (**C**), the dashed line indicates the VIP = 1 threshold.

Principal component analysis of all detected spectrometric features revealed a clear separation among the four experimental groups (Figure 4A). PC1 explained 42.5% of the variance and primarily discriminated against genotypes, whereas PC2 explained 10.3% and separated irrigation treatments within each genotype. A similar separation pattern was observed when the analysis was restricted to the 209 annotated metabolites (Figure 4B; Table S1). In this analysis, PC1 explained 49.3% of the variance while PC2 explained 17.9%, again separating genotypes and water treatments, respectively.

The biplot loading vectors projected from the origin represent the annotated metabolites contributing most strongly to sample discrimination. The metabolite identities corresponding to the loading vectors are provided in Figure 4B and Table S1, while their direction and magnitude reflect associations with genotype- and treatment-dependent metabolic variation.

PLS-DA confirmed and strengthened the separation patterns observed in PCA (Figure 4C). Specifically, the first latent component (t1; 49.3% explained variance) primarily separated the genotypes, whereas the second component (t2; 17.9%) distinguished irrigation treatments within each genotype. Permutation testing confirmed the robustness of the PLS-DA model, which exhibited good explanatory power (*R*^2^*Y* = 0.664), moderate predictive ability (*Q*^2^ = 0.342), and low estimation error (RMSEE = 0.29). The significant permutation-derived *p*-values (*pR*^2^*Y* = 0.001 and *pQ*^2^ = 0.002) further supported that the observed class separation was unlikely to have occurred by chance.

The VIP score plot and associated heatmap (Figure 4D) identified metabolites contributing most strongly to sample discrimination, all presenting VIP scores > 1. These metabolites belonged to several chemical classes, including flavonoids, isoflavones, phenolic acids, amino acids, nucleosides, carbohydrates, and organic acids. Complete metabolite identities, PubChem identifiers, annotation scores, and spectral information are provided in Table S2.

### 3.3. Differentially Accumulated Compounds in Response to Water Deficit

**Figure 5:**
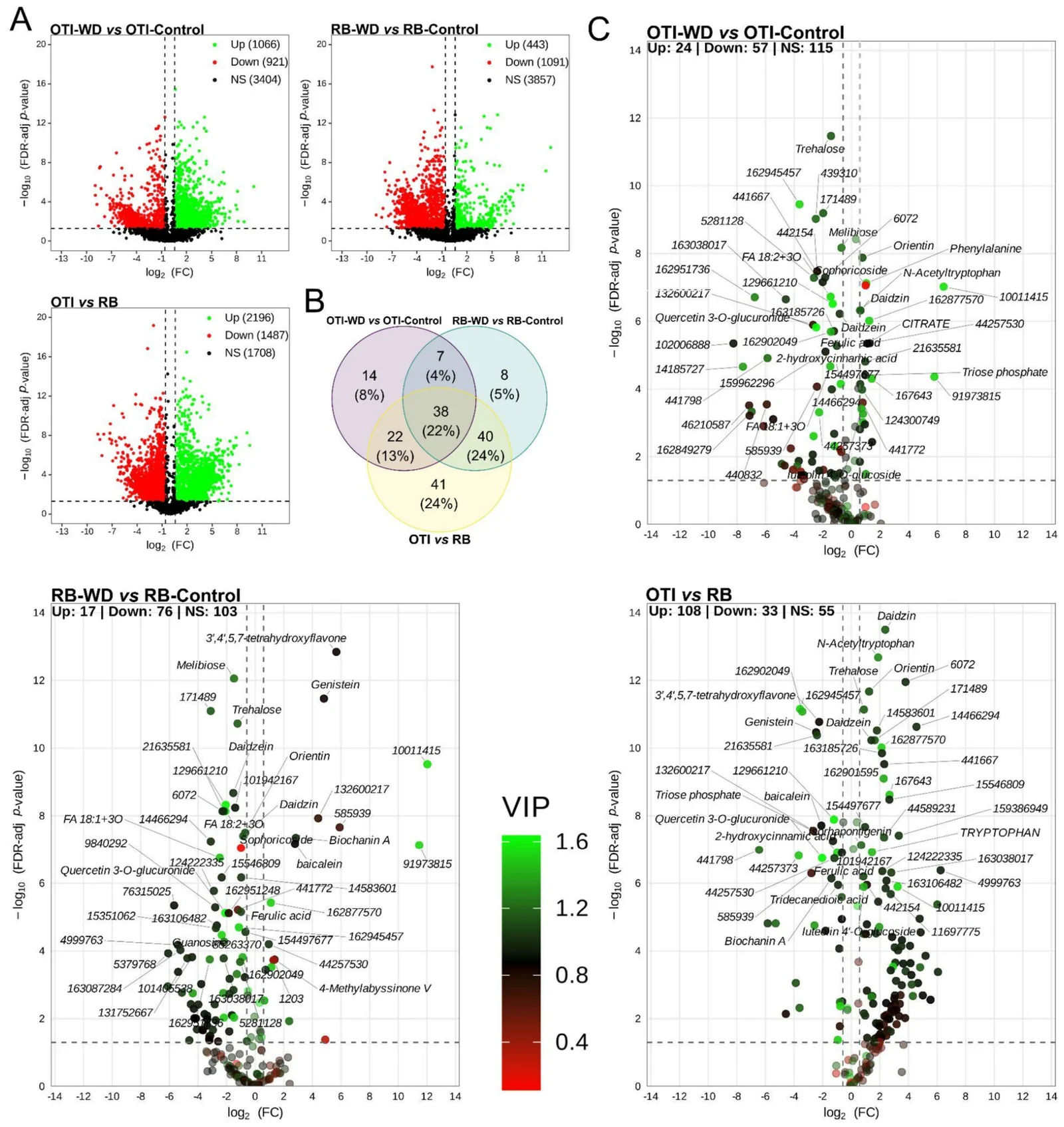
Differential metabolite accumulation analysis. (**A**) Volcano plots highlighting over-accumulated (green) and under-accumulated (red) features in genotype and treatment comparisons. (**B**) Venn diagram showing shared and unique differential metabolites. (**C**) Volcano plots with identified metabolites. The vertical dashed lines indicate the fold-change threshold, and the horizontal dashed line indicates the statistical significance threshold.

Volcano plot analysis revealed clear differences in regulation among comparisons (Figure 5A). In both drought treatment comparisons (OTI-WD vs. OTI-Control and RB-WD vs. RB-Control), the predominant response was a net down-accumulation of metabolites, consistent with the broader feature level trends observed for spectrometric features. In OTI-WD compared with OTI-Control, 1066 features were significantly over-accumulated and 921 were down-accumulated. In RB-WD compared with RB-Control, 443 features were accumulated and 1091 were accumulated. In the genotype comparison (OTI vs. RB), 2196 were over-accumulated and 1487 were down-accumulated (Figure 5A).

Venn diagram analysis further illustrated the overlap and uniqueness among comparisons (Figure 5B). Thirty-eight differentially accumulated metabolites were shared across all comparisons. In OTI-WD vs. OTI-Control, fourteen (8%) were uniquely accumulated, while eight (5%) were exclusive to RB-WD vs. RB-Control. In the genotype comparison, 41 (24%) were uniquely differential.

In addition, volcano plot analysis of identified metabolites revealed distinct differential accumulation profiles across the three comparisons (Figure 5C). In all cases, the predominant response net down-accumulation of metabolites under water deficit, consistent with the broader feature-level trends described above. Only annotated metabolites meeting the significance thresholds (FDR-adjusted *p* < 0.05 and fold change > 1.5) were displayed in Figure 5C.

In OTI (OTI-WD vs. OTI-Control), 24 metabolites were significantly up-accumulated and 57 were down-accumulated. The down-accumulated fraction was dominated by flavonoid glycosides and isoflavones, phenolic acids (ferulic acid, chlorogenic acid derivatives), hydroxycinnamic acid derivatives, and anthocyanin glycosides. Among the 24 up-accumulated metabolites, the most prominent compounds were four highly glycosylated triterpenoid saponins, together with several flavonoids and isoflavones.

In RB, the water restriction response was markedly more restrained, with only 17 metabolites up-accumulated and 76 down-accumulated. The down-accumulated fraction was dominated by flavonoids and their glycosides (including flavones, flavanones, isoflavones, and anthocyanins), as well as glycosylated triterpenoid saponins. In contrast, the up-accumulated fraction was notably enriched in flavonoids and isoflavones, including genistein, biochanin A, baicalein, and phaseol. However, the comparison between genotypes (OTI vs. RB), performed by integrating samples from both irrigation regimes, revealed the greatest metabolic divergence, with 108 metabolites significantly more abundant in OTI and only 33 in RB (Table S2). The OTI xylem sap metabolome was broadly enriched in flavonoids and their glycosides, particularly flavones, flavanones, isoflavones, and anthocyanin derivatives. In contrast, RB xylem sap was enriched in a distinct set of flavonoids and isoflavones, together with several glycosylated triterpenoid saponins.

### 3.4. Metabolic Profiling of Xylem Exudates Under Water Deficit

**Figure 6:**
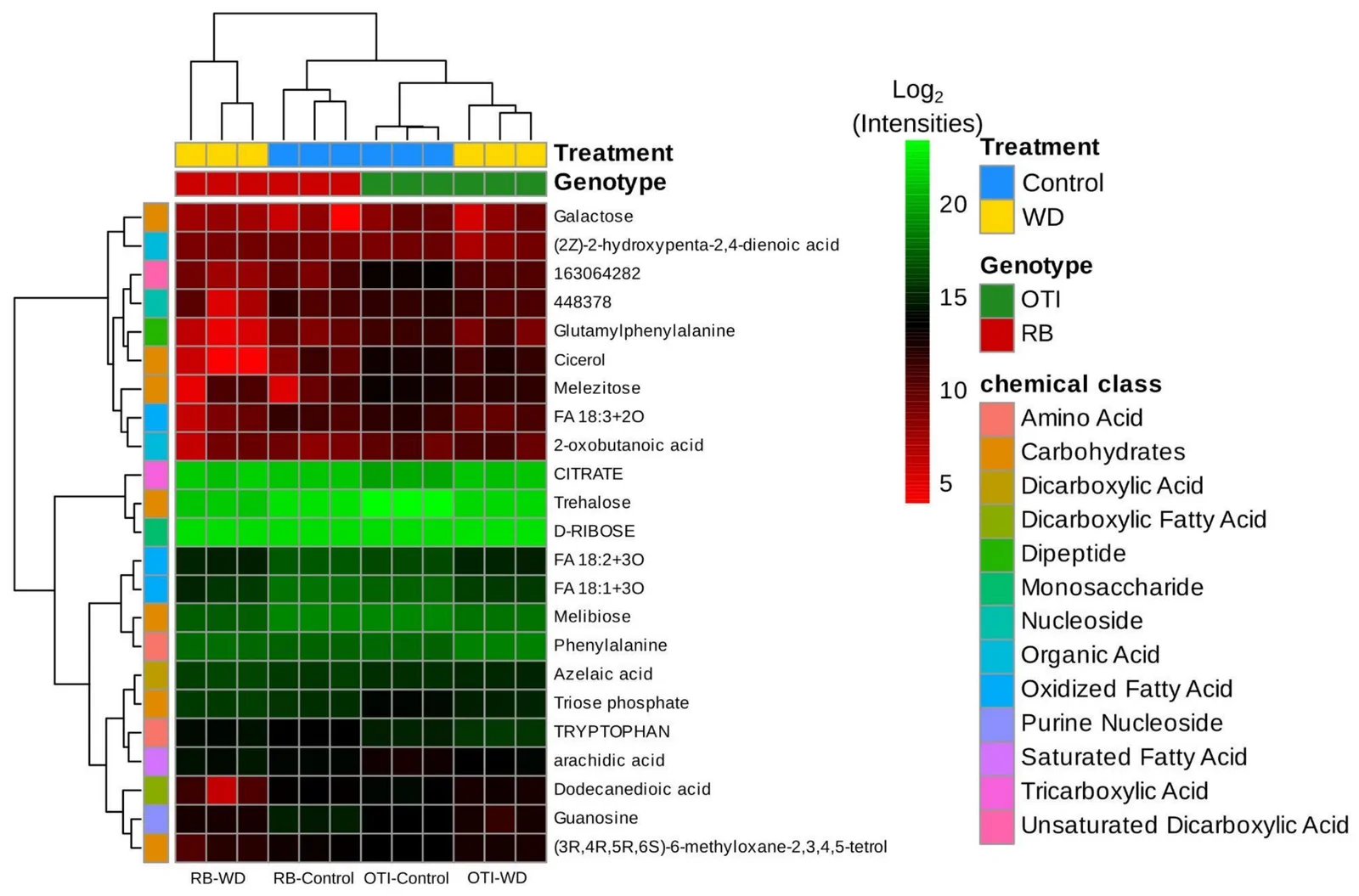
Hierarchical clustering of differentially accumulated xylem metabolites across genotypes and treatments. Heatmap showing the relative abundance of manually curated metabolites associated with primary metabolism in xylem exudates of common beans under control and water deficit conditions. The dendrograms represent hierarchical clustering of both samples (columns) and metabolites (rows) based on similarity in metabolite abundance profiles. Metabolites were grouped by chemical class.

The heatmap in Figure 6 illustrates the relative abundance of key xylem metabolites across the four treatment combinations (OTI-Control, OTI-WD, RB-Control, and RB-WD). Metabolites included in this analysis were manually curated and selected based on their established roles in primary metabolism, including amino acids, organic acids, carbohydrates, fatty acids, and other related compounds. Hierarchical clustering based on metabolite abundance patterns revealed clear separation according to the genotype and irrigation treatment. Amino acids, organic acids, sugars, fatty acids, and other related metabolites exhibited distinct accumulation patterns, with both increases and decreases observed under water deficit conditions (Figure 6).

In RB plants exposed to water deficit, decreases were observed in soluble sugars and energy-related metabolites, including trehalose, melibiose, ciceritol, and citrate, together with reductions in the purine nucleoside guanosine. In addition, several oxidized and dicarboxylic fatty acid (FA 18:1+3O, FA 18:2+3O, FA 18:3+2O, dodecanedioic acid and CID: 5281128) derivatives also showed lower abundance in RB under stress. In contrast, the OTI genotype displayed a comparatively attenuated metabolic reduction under water deficit. Although some metabolites decreased in abundance relative to well-watered controls, compounds involved in osmotic adjustment and central metabolism, including trehalose, myo-inositol, and aromatic amino acids, remained relatively more abundant than in RB plants subjected to the same stress condition.

Overall, the majority of metabolites displayed decreased accumulation under water deficit compared to control conditions. Figure S2 further confirmed that water deficit was the primary factor associated with metabolic variation, characterized by a generalized decrease in metabolite abundance and genotype-specific clustering patterns.

### 3.5. Pathway and Chemical Enrichment Analysis of Xylem Metabolites Under Water Deficit

KEGG pathway enrichment point plots showed the enrichment of different metabolites in metabolic pathways. In the OTI-WD vs. OTI-Control comparison, significant enrichment was observed for stilbenoid, diarylheptanoid, and gingerol biosynthesis (up); phenylalanine metabolism (down); isoflavones biosynthesis (up and down); flavonoid biosynthesis (up); anthocyanin biosynthesis; and indole alkaloid biosynthesis. In the RB-WD vs. RB-Control comparison, fewer pathways reached significance, primarily isoflavonoid biosynthesis (up and down), flavonoid biosynthesis (up and down), flavone and flavonol biosynthesis (up), and anthocyanin biosynthesis (up and down). The genotype comparison (OTI vs. RB) revealed broader pathway differentiation, with isoflavonoid biosynthesis (RB-up), phenylpropanoid biosynthesis (OTI-up), anthocyanin biosynthesis (OTI-up), and flavonoid biosynthesis (OTI-up) among the most prominently enriched pathways, suggesting constitutive metabolic differences between genotypes independent of water treatment.

### 3.6. ChemRICH Chemical Similarity Enrichment Analysis of Significantly Altered Metabolites in Xylem Sap

**Figure 7:**
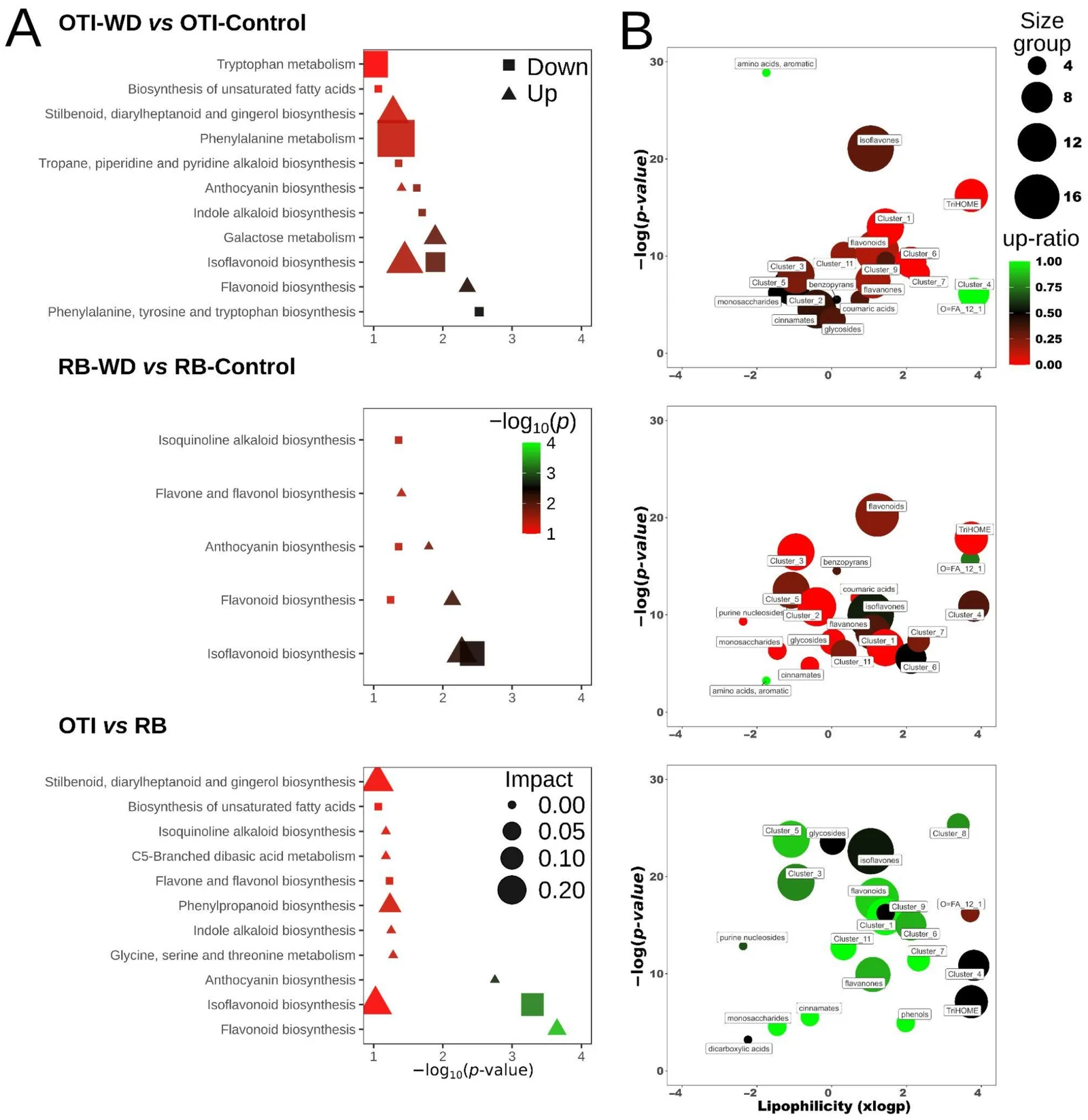
KEGG pathway and ChemRICH enrichment analyses of differentially accumulated metabolites. (**A**) Bubble plots showing pathway significance and impact; networks highlight enriched pathways and associated metabolites. ChemRICH analysis of xylem metabolites under water deficit, showing chemically enriched clusters based on structural similarity and significance (**B**).

Metabolites were grouped according to their structural similarity and visualized according to lipophilicity (xlogP) and enrichment significance (−log FDR-adjusted) (Figure 7B). The bubble size represents the cluster size, and the bubble color indicates the relative proportion of up-accumulated (green) and down-accumulated (red) metabolites within each cluster.

In the OTI-WD vs. OTI-Control comparison, several chemical clusters showed significant enrichment, particularly isoflavones, flavonoids, flavanones, and other related phenolic compounds, predominantly in the intermediate-to-high lipophilicity range. Most of these clusters exhibited a predominance of down-accumulated metabolites (red), consistent with a general reduction in secondary metabolite abundance under water deficit. A few clusters, including certain glycosides, aromatic amino acids, and glycosylated triterpenoid saponins (cluster 4), showed higher proportions of up-accumulated metabolites.

In the RB-WD vs. RB-Control comparison, a similar overall pattern was observed, although with generally smaller cluster sizes and lower enrichment significance. The most prominent clusters corresponded to flavonoids, isoflavones, flavanones, benzopyrans, and coumaric acids, again with a predominance of down-accumulated metabolites. In the genotype comparison (OTI vs. RB), clearer constitutive differences were evident. Several clusters associated with flavonoids, flavanones, isoflavones, glycosides, and other related phenolic compounds were enriched and predominantly up-accumulated in OTI (green), whereas other clusters (dicarboxylic acids, TriHOME, O=FA_12_1, glycosides, isoflavones and cluster 9) showed the opposite pattern.

### 3.7. Functional Analysis of the Protein–Metabolite Interactome

**Figure 8:**
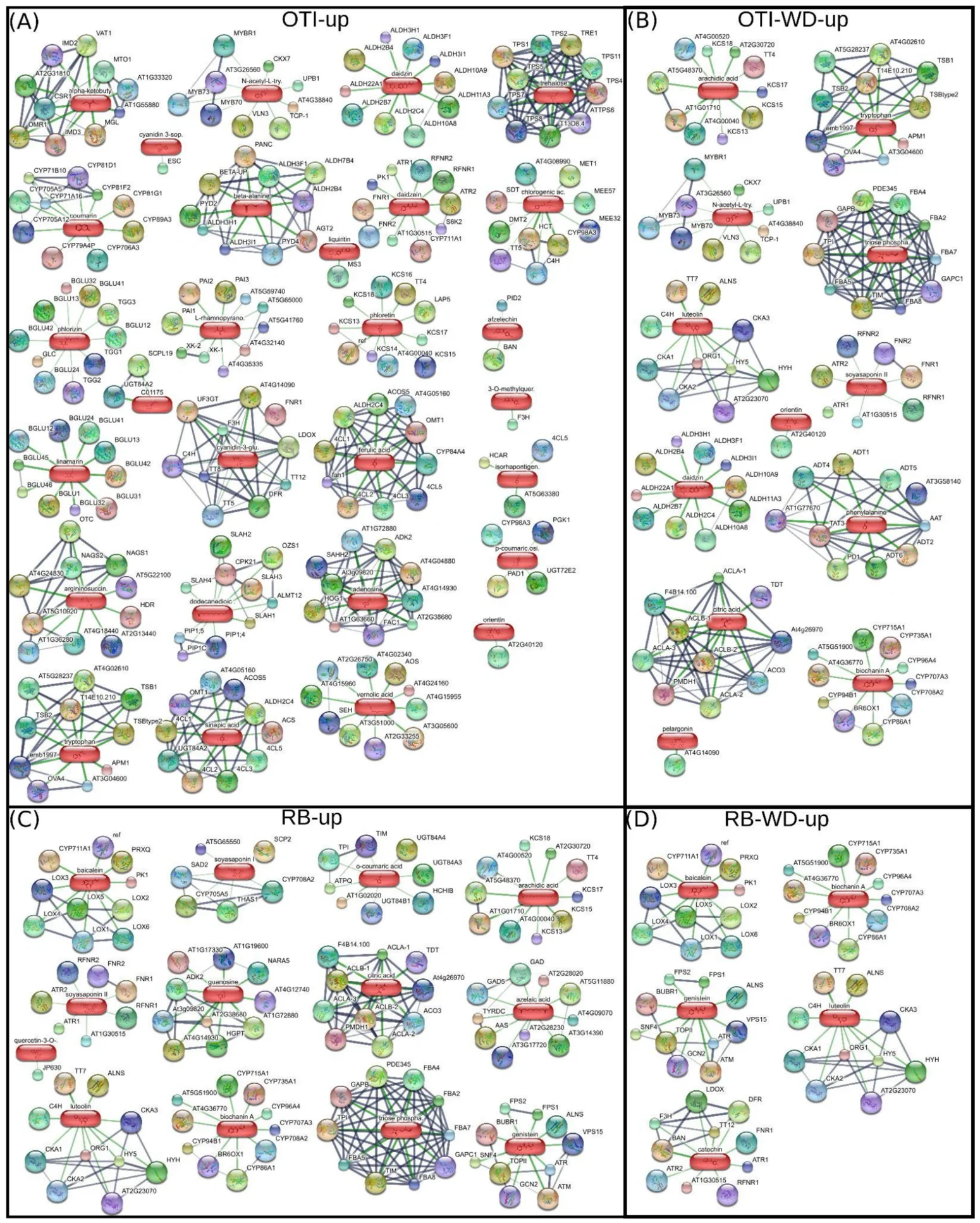
Integrated metabolic network analysis of up-regulated response patterns. STITCH-based network visualizations illustrating the interaction of differentially accumulated metabolites and predicted protein associations for: (**A**) OTI genotype specific markers, (**B**) OTI response to water deficit (WD), (**C**) RB genotype specific markers, and (**D**) RB response to water deficit (WD). Red nodes represent central metabolic hubs, while edges indicate functional or physical associations.

To explore the potential functional relevance of the metabolic shifts detected in xylem sap, up-accumulated metabolites were integrated with predicted protein interactors using STITCH. The resulting interactomes (Figure 8) suggest four genotype- and treatment-dependent sets of modules, pointing to differences in metabolic organization between OTI and RB. Given the exploratory nature of this analysis and reliance on *A. thaliana*-based predictions, these associations should be interpreted as suggestive rather than confirmatory.

Metabolites constitutively enriched in OTI produced an extensive, highly diversified interactome, organized into numerous parallel modules rather than a single hub, consistent with the broader metabolomic profile observed for this long-cycle, tolerant genotype. Major modules linked phenylpropanoid enzymes (C4H, 4CL isoforms, ALDH2C4/OMT1, CYP98A3/HCT) to ferulic, sinapic, and chlorogenic acids and coumarin, as well as flavan-3-ol/anthocyanin enzymes (TT5/8/12, DFR, LDOX) to cyanidin-3-glucoside, broadly matching the daidzin-, liquiritin- and procyanidin-type glycosides enriched in OTI. Additional modules connected beta-glucosidases to linamarin/phlorizin, adenosine kinase/SAHH2/HOG1 to adenosine, and IMD/CSR1/MTO1 to alpha-ketobutyrate, together with smaller trehalose-, tryptophan- and oxylipin-related modules. Collectively, these modules suggest conserved associations with methylation-cycle enzymes, amino acid metabolism, and osmoprotection-related compounds that were maintained independently of water status.

RB-enriched metabolites instead produced a more compact interactome centered on fewer core modules, compatible with this genotype’s shorter, escape-type strategy. The dominant module linked lipoxygenases (LOX1–LOX6) and PRXQ to baicalein, suggesting an association between oxylipin/jasmonate metabolism and redox-related flavonoids. Other modules connected cytochrome P450s to biochanin A and genistein, casein kinases/HY5-HYH to luteolin, and THAS1/P450s to soyasaponins (matching the isoflavone- and saponin-enriched profile of RB), while smaller modules linked UDP-glycosyltransferases to o-coumaric acid and stress/energy kinases (ATM/ATR, GCN2, SNF4) to genistein.

Under water deficit, the OTI interactome displayed additional modules involving citrate metabolism (ATP-citrate lyase subunits and dehydrogenases) together with glycolytic and triose-phosphate metabolism-related genes, potentially extending the range of carbon metabolism-associated interactions observed under control conditions. Aldehyde dehydrogenases also formed connections with daidzin, while additional modules linked arogenate dehydratases to phenylalanine and KCS genes to arachidic acid, extending the network toward aromatic amino acid and very-long-chain fatty acid biosynthesis. Modules associated with luteolin, soyasaponin II, and tryptophan-related metabolites were also retained from the constitutive OTI interactome.

The RB water deficit response produced the smallest and least diversified interactome, largely reinforcing modules already present in RB-up, including LOX–baicalein, biochanin A–cytochrome P450, genistein–ATM/ATR/GCN2/SNF4, and luteolin–HY5/HYH associations. The principal addition was a catechin-related flavan-3-ol module involving LDOX, F3H, TT12, BAN and DFR, suggesting a modest expansion of flavonoid-associated interactions under drought.

## 4. Discussion

Hydraulic limitations are known to trigger a cascade of physiological and metabolic responses aimed at maintaining cellular homeostasis and reproductive success under stress. Recently, we demonstrated that the OTI genotype exhibited a stronger recovery of its water status, as reflected by a higher relative water content after rehydration, while RB showed limited recovery [36,46,47]. Therefore, the physiological responses observed here provide an essential framework for interpreting the extensive metabolic reprogramming detected in xylem exudates, suggesting that changes in vascular metabolism constitute an integral component of the systemic response to water deficit during pod filling.

The metabolomic analysis of xylem exudates revealed an extensive effect of water deficit. Multivariate analyses (PCA and PLS-DA) clearly separated both irrigation treatments and genotypes, demonstrating that water deficit not only altered the abundance of individual metabolites but also reshaped the overall organization of the xylem metabolome.

Changes in xylem sap composition have been reported under multiple environmental constraints, including drought, flooding, and nutrient limitation, reflecting alterations in root metabolism and long-distance signaling processes [24,48,49]. In legumes, drought-induced accumulation of ureides and other nitrogen-related compounds further supports the role of vascular tissues in coordinating whole-plant responses to stress [50,51].

The contrasting metabolite profiles detected between OTI and RB further support the existence of genotype-specific strategies for coping with water deficit. The results indicate that water deficit during pod filling induced a systemic contraction of the xylem sap metabolome, with a significant reduction in the number of metabolites under water deficit. OTI and RB displayed contrasting patterns of metabolite accumulation, indicating genotype-dependent metabolic responses. This comparison was consistent between genotypes (OTI vs. RB), revealing great metabolic divergence, with 108 metabolites significantly more abundant in OTI and only 33 in RB. Although the results indicate that, under conditions of water deficit, these metabolites were reduced, previous reports have linked this to antioxidant protection, stress signaling, and cell-wall-associated defense responses, suggesting stronger activation of protective phenolic metabolism in this genotype [52,53].

Furthermore, the differential accumulation of these metabolite classes suggests that drought adaptation in common beans is not governed by a single metabolic program but by a genotype-dependent reconfiguration of vascular metabolic networks that associate with antioxidant properties and participate in redox regulation; their accumulation in xylem exudates may reflect an integrated signaling system that coordinates oxidative stress mitigation beyond local tissue responses [53].

Several metabolites identified in this study may contribute to the preservation of vascular functionality under water deficit. The presence of *O*-coumaric acid and other related phenolic intermediates suggests the modulation of lignin-associated pathways that reinforce cell walls and potentially improve hydraulic stability during drought [24]. Likewise, glycosylated triterpenoid saponins, particularly enriched in RB, have been associated with membrane protection and stress adaptation, whereas oxylipin-related compounds participate in hormonal signaling pathways involving jasmonates and abscisic acid, two central regulators of drought responses [54,55,56]. Together, these observations indicate that drought-induced changes in xylem composition are not merely passive consequences of altered metabolism but likely represent components of an active vascular response integrating antioxidant defense, structural maintenance, and stress signaling.

The analysis showed that several amino acids and soluble carbohydrates, including L-Rhamnose, melibiose, ciceritol, trehalose, tryptophan, and phenylalanine, exhibited changes in abundance under drought conditions, indicating substantial alterations in carbon allocation, nitrogen metabolism, and osmotic regulation. Because these compounds play central roles in primary metabolism and stress-responsive signaling, their altered accumulation likely reflects a strategic reallocation of resources toward essential maintenance processes and reproductive survival rather than sustained metabolite transport through the vascular system.

Reduced water availability limits photosynthetic carbon assimilation and restricts the availability of substrates required for growth and biosynthetic activity. The increase in aromatic amino acids, such as phenylalanine and tryptophan, may reflect both biosynthetic activity and increased utilization of these compounds as precursors for stress-associated secondary metabolites. Similar reductions in tricarboxylic acid cycle intermediates and amino acid pools have also been reported in vascular tissues of drought-stressed plants, reflecting constraints on central carbon metabolism and nitrogen assimilation [24].

Pathway enrichment analyses provided further evidence that drought-induced metabolic reprogramming in the xylem is organized at the network level rather than through isolated changes in individual metabolites. KEGG enrichment consistently highlighted the modulation of flavonoid and isoflavonoid biosynthesis among the most significantly affected pathways, while ChemRICH analysis revealed coordinated enrichment of metabolite classes associated with phenylpropanoid-derived metabolism. Together, these results indicate that water deficit triggers a structured reorganization of vascular metabolic networks centered on secondary metabolism. This supposition is supported by previous reports where flavonoids are widely recognized for their ability to scavenge reactive oxygen species, regulate cellular redox balance, and modulate stress-responsive signaling pathways [53,57].

Beyond their antioxidant functions, phenylpropanoid-derived metabolites contribute to structural adaptation during drought. Several intermediates identified in this study are associated with lignin biosynthesis and cell wall remodeling, processes that may enhance vascular stability and maintain hydraulic functionality under reduced water availability. The reinforcement of vascular tissues through phenolic metabolism has been proposed as an important component of drought acclimation, as it increases resistance to mechanical stress and may reduce the vulnerability of conductive tissues to hydraulic dysfunction [58,59,60]. The simultaneous enrichment of metabolites associated with antioxidant defense and structural reinforcement therefore suggests that phenylpropanoid metabolism contributes to both biochemical and physical resilience during water deficit.

Drought stress has been shown to stimulate the accumulation of flavonoids, phenolic acids, and other phenylpropanoid-derived compounds in multiple tissues of *P. vulgaris*, including roots, leaves, and reproductive organs [60,61]. Metabolomic and transcriptomic analyses have likewise identified flavonoid biosynthesis, phenylpropanoid metabolism, and the associated antioxidant pathways among the most responsive components of drought adaptation in common beans and other related species [26,62]. Similar enrichment patterns have been reported in soybean and alfalfa, where the activation of flavonoid and isoflavonoid pathways contribute to enhanced tolerance through improved redox regulation and stress acclimation [63,64].

The combined metabolomic, pathway enrichment, ChemRICH, and interactome analyses revealed that each genotype deployed a distinct vascular response architecture, indicating that drought adaptation is governed by alternative configurations of metabolic regulation rather than by uniform changes in metabolite abundance.

OTI exhibited a broad and metabolically diverse response characterized by the accumulation of isoflavones, phenolic derivatives, amino acid related compounds, and metabolites associated with detoxification and stress signaling. KEGG and ChemRICH analyses consistently highlighted the enrichment of phenylpropanoid-derived pathways, including flavonoid, isoflavones, anthocyanin, and aromatic amino acid metabolism, suggesting extensive activation of protective secondary metabolism. The simultaneous enrichment of these interconnected pathways may reflect a coordinated acclimation program that integrates antioxidant defense, metabolic flexibility, and vascular maintenance during prolonged water deficit.

Particularly noteworthy was the presence of aldehyde detoxification networks centered on glyceraldehyde-associated metabolites and aldehyde dehydrogenase-related interactions, which may be associated with the removal of reactive carbonyl compounds generated during oxidative stress. The maintenance of hydroxycinnamic acid derivatives and other phenylpropanoid intermediates further indicates the persistence of phenylpropanoid-related metabolic activity under reduced water availability, although their specific roles in vascular integrity and hydraulic protection require further investigation [24,60,63,65].

The interactome analysis revealed that drought did not induce comparable network expansion in RB. Instead, the response was characterized by reinforcement of pre-existing modules centered on soyasaponins, flavonoids, and lipoxygenase-associated subnetworks. Soyasaponin I and soyasaponin II emerged as major hubs within the RB interactome, constitutive with a prominent role for these triterpenoids in vascular stabilization and stress protection under drought. Likewise, the prominence of LOX-associated subnetworks, including multiple lipoxygenase-related nodes, points to enhanced oxylipin-mediated signaling. Because lipoxygenase activity contributes to jasmonate biosynthesis and stress induced developmental transitions, including senescence and nutrient remobilization [56,65], this network organization is consistent with a link between oxylipin signaling and nutrient remobilization processes in RB—a pattern that should be confirmed with additional biological replicates.

Future integration of xylem metabolomics with complementary omics approaches will help elucidate the regulatory mechanisms underlying vascular metabolic reprogramming and their contribution to drought adaptation.

## 5. Conclusions

The effects of water deficit during pod filling are associated with a systemic metabolic contraction in the xylem sap of *P. vulgaris* and characterized by genotype-specific response patterns. The long-cycle genotype, OTI, exhibited a broader metabolic response plasticity through the accumulation of aromatic amino acids, citrate, isoflavones, and flavonoid glycosides, which may support oxidative stress management and carbon remobilization. In contrast, the early-maturing RB genotype potentially relies on a more specialized and compact defense network involving flavonoids, isoflavones, and glycosylated triterpenoid saponins. These observations suggest that the reorganization of the vascular metabolome could represent an active response that integrates antioxidant defense may contribute to vascular function. Ultimately, the limitation of this exploratory study is that additional tools are needed to better understand the mechanisms underlying systemic drought adaptation and to identify candidate biomarkers for breeding climate-resilient common bean cultivars.

## Data Availability

The original contributions presented in this study are included in the article and Supplementary Materials. Further inquiries can be directed to the corresponding authors.

## Supplementary Materials

The following supporting information can be downloaded at https://www.mdpi.com/article/10.3390/metabo16080564/s1, Figure S1: The upper panel shows plants of the common bean genotypes OTI and RB grown under well-watered conditions (100% field capacity, FC), whereas the lower panel shows plants subjected to water deficit stress (50% FC) for 10 days. Scale bar = 10 cm. Figure S2: Hierarchical clustering heatmap of identified metabolites between treatments and genotypes. Table S1: Spectrometric information and detailed annotation information for putatively identified metabolites detected in common bean xylem sap by UPLC–ESI–QTOF–MS. Table S2: Differentially accumulated metabolites (DAMs) in response to water deficit and genotypes. Statistical summary of metabolites showing significant changes (FDR-adjusted *p*-value < 0.05) across experimental contrasts: OTI-WD vs. OTI-Control, RB-WD vs. RB-Control, and OTI vs. RB. Table S3: Chemical enrichment analysis of the xylem sap response. Detailed results from ChemRICH chemical similarity clustering analysis.

## Abbreviations

The following abbreviations are used in this manuscript:

ABA	Abscisic Acid
FC	Field Capacity
FDR	False Discovery Rate
GC-MS	Gas Chromatography–Mass Spectrometry
HCA	Hierarchical Clustering Analysis
KEGG	Kyoto Encyclopedia of Genes and Genomes
LC-MS	Liquid Chromatography–Mass Spectrometry
MSI	Metabolomics Standards Initiative
NMR	Nuclear Magnetic Resonance Spectroscopy
OTI	OTI Common Bean Genotype
PCA	Principal Component Analysis
PLS-DA	Partial Least Squares Discriminant Analysis
PTFE	Polytetrafluoroethylene
QTOF	Quadrupole Time-Of-Flight
RB	Rosa La Bufa Common Bean Genotype
RT	Retention Time
STITCH	Search Tool for Interacting Chemicals
UPLC	Ultra-Performance Liquid Chromatography
UPLC–ESI–QTOF–MS	Ultra-Performance Liquid Chromatography Coupled with Electrospray Ionization Quadrupole Time-Of-Flight Mass Spectrometry
VIP	Variable Importance in Projection
WD	Water Deficit
